# A closer association between blood urea nitrogen and the probability of diabetic retinopathy in patients with shorter type 2 diabetes duration

**DOI:** 10.1038/s41598-023-35653-z

**Published:** 2023-06-19

**Authors:** Jian-Bo Zhong, Yu-Feng Yao, Guo-Qiang Zeng, Yi Zhang, Bai-Kang Ye, Xiao-Yan Dou, Li Cai

**Affiliations:** 1 Department of Ophthalmology, Shenzhen University General Hospital, Shenzhen, Guangdong Province China; 2 Shenzhen University Medical College, No. 3688 Nanhai Ave, Shenzhen, 518061 Guangdong Province China; 3 Department of Ophthalmology, Shenzhen Second People’s Hospital, The First Affiliated Hospital of Shenzhen University, Shenzhen, Guangdong Province China; 4 Shantou University Medical College, No. 22 Xinling Road, Shantou, 515031 Guangdong Province China

**Keywords:** Retinal diseases, Diabetes complications, Type 2 diabetes

## Abstract

Blood urea nitrogen (BUN) is an indicator of renal function and catabolic status in human body. Diabetic retinopathy (DR) is a major microvascular complication of diabetes mellitus (DM) and a serious threat to the vision of diabetic patients. We included 426 type 2 diabetic patients who visited the endocrinology department of Guangdong Provincial People’s Hospital and received an ophthalmology consultation from December 2017 to November 2018. The outcome was the probability of DR in participants. Multivariable logistics analysis was used to confirm the relationship between BUN and the probability of DR. And interaction tests were conducted to find the effects of DM duration on their association. A total of 167 of 426 patients with type 2 diabetes had DR, with a probability of 39.20%. After adjusting for potential confounders, a positive association between BUN and the probability of DR (OR = 1.12; 95% CI 1.03–1.21; P = 0.0107). And a test for interaction between DM duration and BUN on the probability of DR was significant (P = 0.0295). We suggested that in patients with type 2 diabetes, BUN was positively associated with the probability of DR and the association was influenced by DM duration.

## Introduction

Diabetic retinopathy (DR) is one of the common microvascular complications of diabetes mellitus (DM) and the leading cause of visual impairment and blindness in the global working-age population^1^. It is predicted that the number of patient with DM will reach 702 million worldwide and 147 million in China by 2045. The prevalence of DR and vision-threatening DR was shown to a range between 28.5% and 33.2% in numerous epidemiological studies^2^. Severe diabetic retinopathy not only imposes a heavy financial burden on medical care, but also seriously threatens the visual quality of patients^3^.

Some researches suggested that BUN could predict DR risk^4^ and was an independent risk factor for proliferative diabetic retinopathy (PDR)^5^. In clinical practice, patients with long-standing diabetes are more susceptible to DR and tend to be severer. According to an American epidemiological survey, the prevalence of DR and PDR in patients with a 5-year DM duration was 28.8% and less than 2.0%, while in patients with a 15-year DM duration, 77.8% and 15.5% respectively^6^. Thus, DM duration is an important risk factor influencing the development of DR. However, there are few studies related to the correlation between BUN or DM duration and the probability of DR. Some studies^5,7,8^ have indicated an association between the probability of DR and BUN, DM duration respectively, but none of them clearly presents the joint effect of BUN and DM duration on DR probability. To address this gap in knowledge, our study aimed to investigate the quantitative relationship between BUN and DR probability in patients with type 2 diabetes, and whether the probability of DR is jointly affected by BUN and DM duration.

## Method

### Data source and study population

We obtained the data from the "Dryad" database (https://datadryad.org). This website allows users to download the raw data from the literature for free. In accordance with the Dryad terms of service, we cited the corresponding Dryad data package in this paper. [Xuenan Zhuang et al.^9^, Data from: Association of diabetic retinopathy and diabetic macular edema with renal function in southern Chinese patients with type 2 diabetes mellitus: a single-center observational study, Dryad, Dataset, https://doi.org/10.5061/dryad.6kg1sd7]. The study was a single-center cross-sectional study that included 426 patients with type 2 diabetes who were hospitalized in the endocrine department of Guangdong Provincial People's Hospital and received an ophthalmic consultation from December 2017 to November 2018. The exclusion criteria were: (1) previous history of intravitreal drug injections or renal dialysis; (2) other eye diseases affecting ocular circulation such as glaucoma, endophthalmitis, retinal vascular obstruction, age-related macular degeneration, refractive error >3.00D, and ocular trauma; (3) severe systemic diseases such as connective tissue disease and cardio-cerebrovascular disease; (4) women in pregnancy or menstrual status. More research details were described in detail in the original^9^. The authors of the original research had clearly stated that: This study was performed according to the Declaration of Helsinki and approved by the Research Ethics Committee of Guangdong Provincial People's Hospital (registration number: gdrec2016232A). All clinical information is obtained through the electronic medical record.

### Variable source and definition of DR

All clinical information was obtained from the electronic medical record. Laboratory tests include liver and kidney function, lipid analysis, and urinalysis. Blood samples and urine samples are obtained when patients were at fasting before 8:00 a.m. DR was diagnosed by a fundus specialist through fundus photography. DR was classified into 5 groups according to the international clinical diabetic retinopathy grading criteria^10^: (1) no significant retinopathy; (2) mild non-proliferative diabetic retinopathy (NPDR); (3) moderate NPDR; (4) severe NPDR. (5) proliferative diabetic retinopathy (PDR). In the study, (2)–(5) were defined as the presence of DR. The measurement and assessment methods and criteria for each variable were described in detail in the original^9^.

### Statistical analysis

The clinical characteristics of the participants were described and divided into two groups according to the presence or absence of DR. For continuous variables with normal distribution, data are presented in the form of “Mean ± SD” with p-values obtained by t-test for two independent samples. For continuous variables with abnormal distribution, data are presented in the form of “Median (Q1-Q3)” with p-values obtained by Mann-Whitney U test. For categorical variables, data are presented as in the form of “sample size (%)” with p-value obtained by χ^2^ test.

The logistic regression model was constructed to analyze the association between BUN and the probability of DR. Firstly, BUN was analyzed as a continuous variable, and then BUN was divided into four groups according to quartiles to further verify the association between BUN and the probability of DR. We presented the unadjusted, minimally adjusted and fully adjusted models according to the recommendations of STROBE statement. Covariates need to be adjusted when they met the following three criteria: (1) Covariate when was included or excluded from the model, the odd ratio changes by at least 10%^11^; (2) Covariate was associated with both BUN and the probability DR based on clinical practice; and (3) Covariate was adjusted in previous similar studies^12^. Curve fitting and interaction tests were used to assess the effect of DM duration on the association between BUN and the probability of DR. In all analyses, P values less than 0.05 (two-sided) were considered statistically significant. The software for statistical analysis of the data was EmpowerStats version 4.1 (www.empowerstats.net, X&Y solutions, Inc. Boston, Massachusetts) and the R language package version 4.2.0 (The R Foundation; http://www.r-project.org; version 4.2.0).

## Results

### The characteristic of participants

The clinical characteristics of the study participants are shown in Table 1. A total of 426 patients (240 males and 186 females) participated in the study with a mean age of 59.00 ± 13.42 years and a median DM duration of 10 years (range: 1–31 years). 167 participants had DR, with a probability of 39.20%. The probability of mild, moderate, severe NPDR and PDR was 8.92% (n = 38), 15.96% (n = 68), 7.75% (n = 33) and 6.57% (n = 28) respectively. Compare with patients without DR, patient with DR had a lower proportion of males, longer DM duration, lower BMI, lower serum levels of ALB, ALT, AST, higher levels of BUN and D-dimer, and higher probability of hypertension, dyslipidemia, renal insufficiency and DME (Table 1).

**Table 1:** Characteristics of the Study Participants.

Variable	Without DR (n = 259)	With DR (n = 167)	P-value
Age (years)*	58.08 ± 13.70	60.41 ± 12.90	0.081
Male sex, n (%)#	162 (62.55%)	78 (46.71%)	0.001
DM duration (years)‡	6.00 (1.00–12.00)	10.00 (7.50–18.00)	< 0.001
BMI (kg/m2)‡	25.1 (22.8–27.0)	24.0 (22.2–26.5)	0.016
SBP (mmHg)‡	133.00 (124.00–147.00)	140.00 (125.50–159.50)	< 0.001
DBP (mmHg)*	80.22 ± 11.60	79.60 ± 11.49	0.588
Hypertension, n (%)#	114 (44.02%)	94 (56.29%)	0.013
Serum albumin (g/L)‡	38.20 (36.30–40.10)	36.90 (34.40–39.70)	0.002
HbA1c (%)‡	9.40 (7.80–11.28)	9.40 (8.00–11.20)	0.987
ALT (U/L)‡	20.00 (15.00–27.00)	16.00 (12.00–23.00)	< 0.001
AST (U/L)‡	19.50 (16.00–24.00)	17.00 (14.00–23.00)	0.002
Acetylcholinesterase (U/L)*	8463.67 ± 2011.18	8150.35 ± 2116.33	0.126
D-dimer (ug/L)‡	330.00 (270.00–502.50)	410.00 (290.00–705.00)	< 0.001
TC (mmol/L)‡	4.80 (3.90–5.60)	5.10 (4.00–6.10)	0.030
TG (mmol/L)‡	1.51 (1.11–2.19)	1.49 (0.96–2.40)	0.929
HDL (mmol/L)‡	0.96 (0.82–1.11)	1.02 (0.85–1.21)	0.027
LDL (mmol/L)*	3.12 ± 0.91	3.33 ± 1.06	0.026
NEFA (mmol/L)‡	0.39 (0.26–0.53)	0.32 (0.20–0.45)	< 0.001
Lipoprotein A (mg/L)‡	112.50 (60.00–246.00)	134.00 (82.00–262.00)	0.032
Apolipoprotein A (g/L)‡	1.14 (1.01–1.26)	1.15 (1.01–1.31)	0.287
Apolipoprotein B (g/L)‡	0.92 (0.74–1.09)	0.97 (0.75–1.17)	0.095
BUN (mmol/L)‡	5.37 (4.31–6.74)	6.13 (4.90–8.64)	< 0.001
Serum creatinine (umol/L)‡	72.00 (62.40–85.75)	75.60 (62.88–103.60)	0.032
Urinary albumin (mg/L)‡	6.77 (3.27–18.26)	28.71 (6.10–251.66)	< 0.001
Urinary creatinine (umol/L)‡	8.23 (5.09–13.34)	5.87 (3.92–8.31)	< 0.001
UACR (mg/g)‡	5.83 (3.24–17.51)	41.09 (9.64–295.49)	< 0.001
eGFR (mL/min/1.73 m^2^)‡	92.58 (75.89–103.46)	80.09 (48.28–98.23)	< 0.001
UACR Stage (≥ Stage 3), n (%)#	11 (4.25%)	51 (30.54%)	< 0.001
CKD Stage (≥ Stage 3), n (%)#	29 (11.20%)	52 (31.14%)	< 0.001
With DME, n (%)#	0 (0.00%)	54 (32.34%)	< 0.001

*DR* diabetic retinopathy, *DM* diabetes mellitus, *SBP* systolic blood pressure, *DBP* diastolic blood pressure, *HbA1c* hemoglobin A1c, *ALT* alanine aminotransferase, *AST* aspartate transaminase, *TC* total cholesterol, *TG* triglycerides, *HDL* high-density lipoprotein, *LDL* low density lipoprotein, *NEFA* non-estesterified fatty acid, *BUN* blood urea nitrogen, *UACR* urine albumin-to-creatinine ratio, *eGFR* estimated glomerular filtration rate, *CKD* chronic kidney disease, *DME* diabetic macular oedema.

*For continuous variables with normal distribution, values were presented as Mean ± SD.

‡For continuous variables with abnormal distribution, values were presented as Median (Q1–Q3).

#For categorical variable, values were presented as N(%).

### The association between BUN and the probability of DR

**Figure 1:**
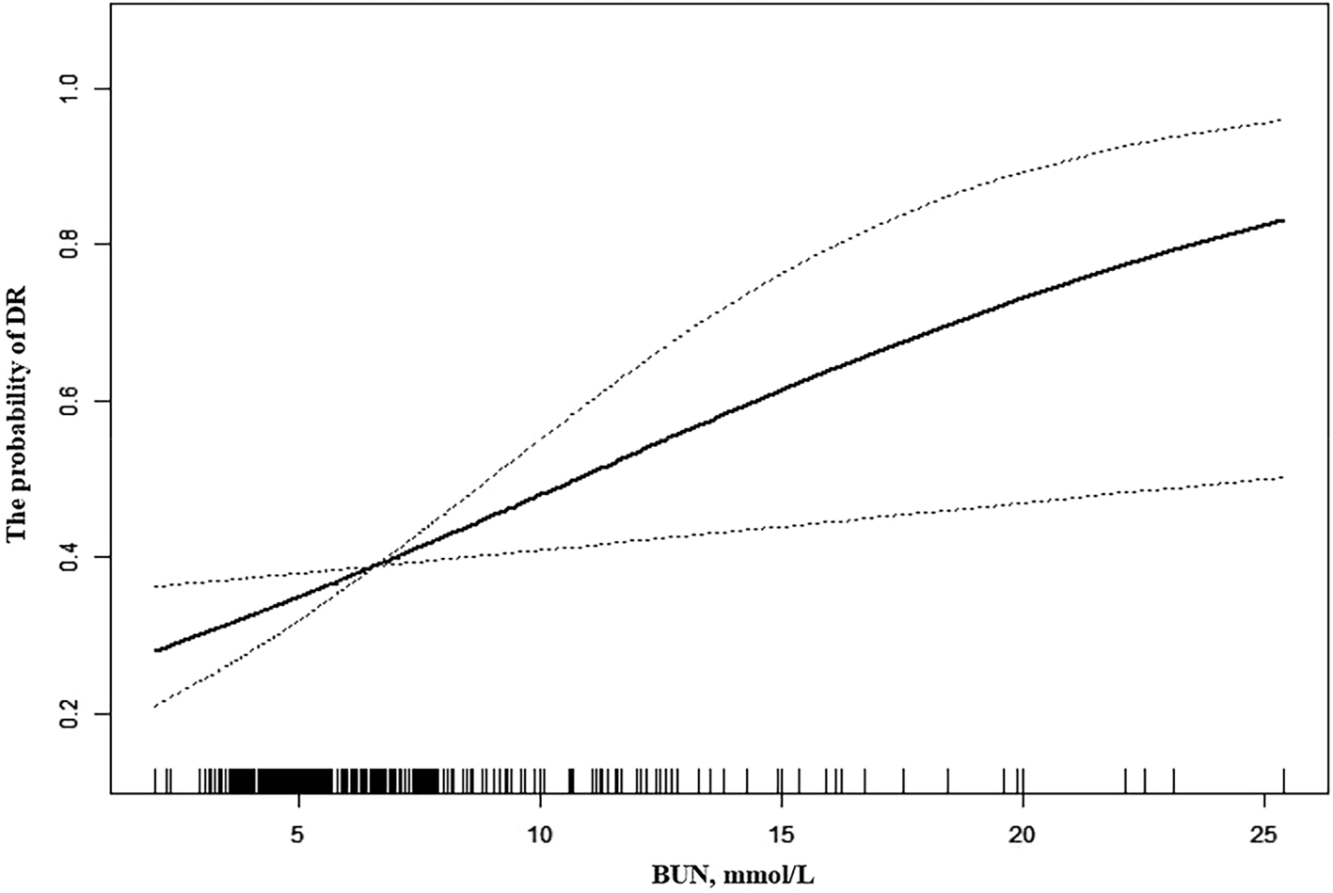
The association between BUN and the probability of DR. A linear association between them was detected after adjusting for age, sex, DM duration, D-dimer, LDL, HbA1c, serum albumin, HBP, SBP. The dashed line indicates 95% CI.

We constructed logistic regression models to assess the relationship between BUN and the probability of DR. In the crude model, BUN was positively associated with the probability of DR (OR = 1. 18, 95% CI 1.10–1.26, P < 0.0001). In the minimally adjusted model, the OR was 1.18 (95% CI 1.09–1.27, P < 0.0001). In the fully adjusted model, the probability of DR was increased by 12% for each 1 mmol/L increased in BUN (OR = 1.12, 95% CI 1.03–1.21, P = 0.0107. Table 2). For sensitivity analysis, we handled BUN as a categorical variable (quartiles) and found that the trend of increase was not significant in the fully adjusted model (P = 0.0522, Table 2). The curve fit plot of fully adjusted BUN and DR shows a linear relationship (Fig. 1).

**Table 2:** The linear association between BUN and the probability of DR in different models.

Variable	Crude model	Minimally adjusted model	Fully adjusted model
BUN	1.18 (1.10, 1.26) < 0.001	1.18 (1.09, 1.27) < 0.001	1.11 (1.03, 1.21) 0.011
BUN (Quartile)			
Q1	Reference	Reference	Reference
Q2	1.67 (0.93, 3.00) 0.087	1.70 (0.91, 3.15) 0.094	1.82 (0.95, 3.48) 0.070
Q3	1.56 (0.88, 2.79) 0.131	1.58 (0.85, 2.94) 0.150	1.64 (0.84, 3.18) 0.144
Q4	3.27 (1.85, 5.77) < 0.001	3.35 (1.75, 6.42) 0.001	2.14 (1.06, 4.31) 0.033
P for trend	0.001	0.001	0.053

The models were presented as OR (95%CI) P-value.

Crude model: we did not adjust other covariants.

Minimally adjusted model: we adjusted age, sex and DM duration.

Fully adjusted model: we adjusted age, sex, DM duration, D-dimer, LDL, HbA1c, serum albumin, HBP, SBP.

### Statistical interaction between BUN and DM duration on the probability of DR

**Figure 2:**
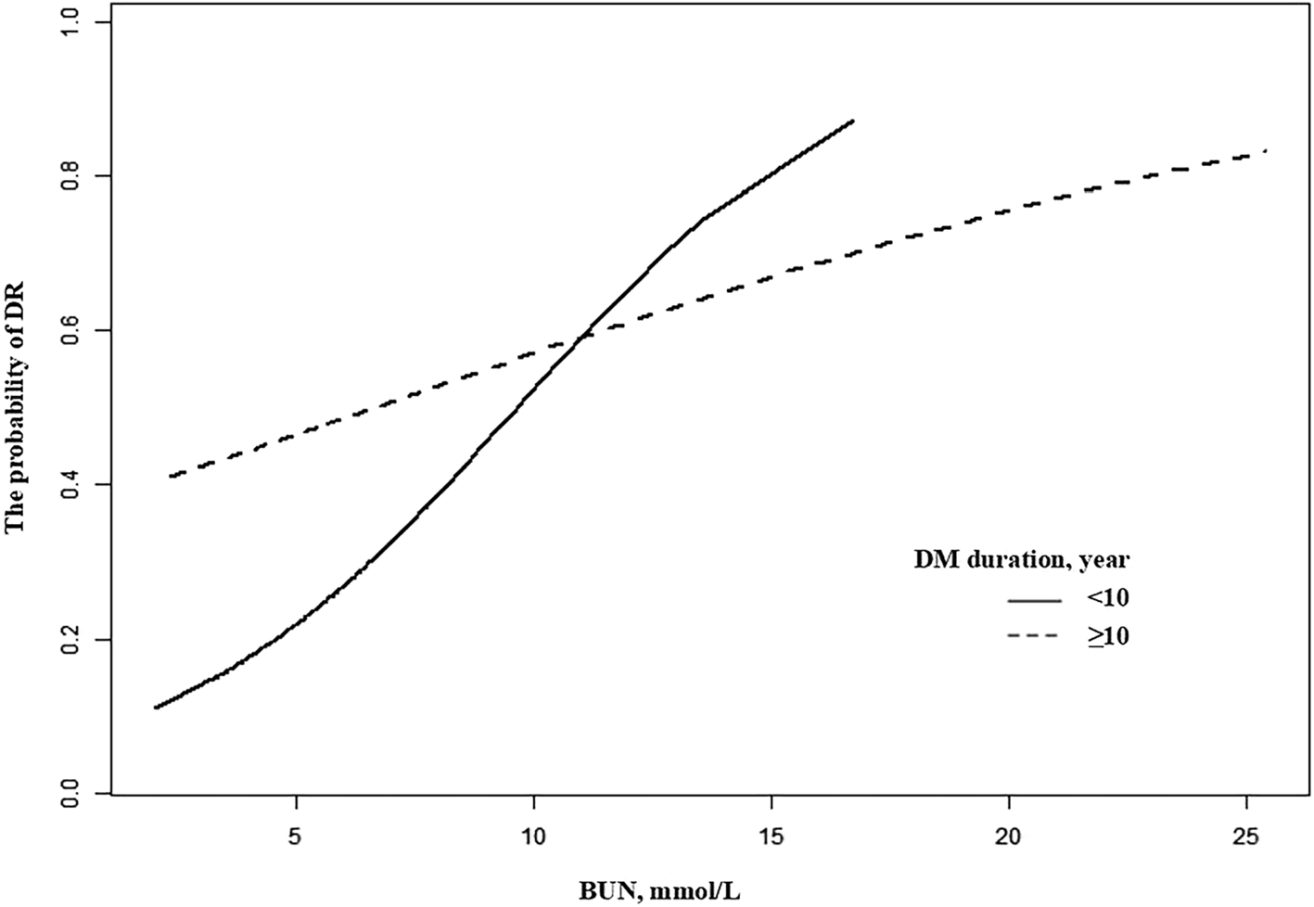
Smooth curves between BUN and the probability of DR stratified by DM duration. The graph displays the adjusted association between BUN and the probability of DR stratified by DM duration. The model adjusted for age, sex, D-dimer, LDL, HbA1c, serum albumin, HBP, SBP.

DM duration was divided into long DM duration group (DM duration ≥ 10 years) and short DM duration group (DM duration < 10 years) based on the median disease duration (10 years). Figure 2 presents the association between BUN and the probability of DR stratiied by DM duration. And both groups shows the same trend of increase. However, the rising trend in short DM duration was significantly faster than long DM duration (Fig. 2). The interaction test was performed and the results were statistically significant in crude model, minimally adjusted model and fully adjusted model with the stable and consistent OR values (P = 0.0306, 0.0092, 0.0295 respectively; Table 3), which suggested that the association between BUN and the probability of DR in patients with type 2 diabetes may be influenced by DM duration.

**Table 3:** Effect modification of DM duration on the relationship between BUN and the probability of DR in different models.

	N	OR (95%CI) P-value	P for interaction
Crude model			
BUN ‡ DM duration < 10 years	208	1.31 (1.13, 1.52) 0.001	0.031
BUN ‡ DM duration ≥ 10 years	216	1.10 (1.02, 1.18) 0.013	
Minimally adjusted model			
BUN ‡ DM duration < 10 years	206	1.43 (1.21, 1.69) < 0.001	0.009
BUN ‡ DM duration ≥ 10 years	216	1.13 (1.05, 1.22) 0.002	
Fully adjusted model			
BUN ‡ DM duration < 10 years	198	1.31 (1.10, 1.57) 0.003	0.048
BUN ‡ DM duration ≥ 10 years	206	1.09 (1.00, 1.19) 0.051	

Crude model: we did not adjust other covariants.

Minimally adjusted model: we adjusted age and sex.

Fully adjusted model: we adjusted age, sex, D-dimer, LDL, HbA1c, Serum albumin, HBP, SBP.

## Discussion

Our results showed that BUN was positively associated with the probability of DR in patients with type 2 diabetes after adjusting for potential covariates, which is consistent with previous studies. And we found that the relationship was influenced by DM duration.

Some researches had reported the positive association between BUN and the probability of DR. Our results were similar to a cross-sectional study of patients with type 2 diabetes finished by Wu et al.^5^. They got the odd ratio between BUN and the probability of DR by a multifactorial logistic regression model in 298 patients and p-values remained significant after adjusting for potential covariates, which suggested that BUN was associated with the probability of DR in individuals with type 2 diabetes^5^. However, covariates were only adjusted for age and smoking history in their work, with the lack of other potential covariate such as hypertension, serum albumin and so on^13–15^. Tan et al. also reported similar findings, but covariate was adjusted only for age^16^. Moreover, Zhang et al. found a positive correlation between BUN and the prevalence of DR in a type 2 diabetic population, which was similar to the results of our study^12^. However, they did not further clarify the specific relationship between BUN and the prevalence of DR by other analysis methods such as curve fitting or interaction test as our work^12^.

However, the mechanism of the association between BUN levels and the prevalence of DR in type 2 diabetic patients is not yet clear. BUN levels are often elevated in DM patients with diabetes nephropathy (DN), suggesting that BUN may be an important indicator of impaired renal function and disturbed glucose homeostasis^17^. Prolonged hyperglycemia triggered excessive oxidative stress, and subsequently leaded to inflammation and microvascular endothelial dysfunction, which is the common pathophysiological mechanism of DN and DR^18^. And either of them may develop along with the other^19–23^. Therefore, elevated levels of BUN tended to suggest the presence of DN or DR^8^. Moreover, BUN was closely related to the catabolic activity in human body, thus its elevated levels may reflect the decrease of circulation and the status of hypercoagulable or oxidative stress^24^. And microvascular hypoperfusion and oxidative stress were important mechanisms in the development of DR^25^, which may account for the elevated levels of BUN in patients with DR.

Investigating subgroup analysis is extremely important for scientific studie^11^. Unfortunately, previous researches tended to performed partial subgroup analyses rather than tests for interactions. It would hinder the exploration to the true relationship between BUN and the probability of DR^7^. In subgroup analysis of our study, the participants were divided into two groups according to the median level of DM duration (10 years)^13^ and we found that BUN was positively associated with the probability of DR in both groups. Figure 2 shows that the probability of DR in short DM duration group (< 10 years) grew faster than that in long DM duration group (≥ 10 years) although the latter had a higher DR probability. It suggested that the association between BUN and the probability of DR may be influenced by DM duration — a closer association was observed in patients with shorter DM duration^26^. The findings could be explained by the insensitivity to BUN changes caused by microvascular endothelial dysfunction^27^ and prolonged hyperpermeability^18,28^ in patients with long DM duration. And R Kawasaki et al. also found that the incidence of DR increased rapidly in type 2 diabetic patients with a DM duration from 5 to 10 years^29^. It may remind us to strengthen the management and follow-up of patients with DM duration less than 10 years and actively intervene in the BUN levels to resist the development of DR. The potential mechanisms among BUN, DM duration and DR need to be elaborated in further researches.

The novelty of our work was that we not only presented the linear association between BUN and the probability of DR in the form of curve fitting, but also found the joint effect of BUN and DM duration on DR probability. Certainly, there were some limitations in our study. First, this study was an analytical cross-sectional study and thus provided only weak evidence between exposure and outcome, making it difficult to elaborate the causality. Second, because the population in our study was southern Chinese patients with type 2 diabetes, our conclusion should be applied with caution in population with other type diabetes or in other regions. Third, we could not observe the association between BUN and other fundus lesions due to the limits of the original data. Lastly, the use of other medications should be considered because of its effect on the development of DR. However, our secondary analysis was based on a publicly available database lacking of the variables about the use of medication, which led to the failure of the adjustment for drug use.

In conclusion, we found a positive association between BUN and the probability of DR in patients with type 2 diabetes, and the association was stronger in patients with a shorter DM. Finally, we must emphasize that our findings are simply hypothesis-generating and further prospective researches were required to confirm our findings.

## Data Availability

The dataset was collected by Sho et al. and is now available on Dryad (via: https://doi.org/10.5061/dryad.fn6730j). The datasets generated or analysed during the current study are available from the corresponding author on reasonable request.
